# Retracted: Biomolecular Modulation of Neurodegenerative Events during Ageing

**DOI:** 10.1155/2019/3571916

**Published:** 2019-10-15

**Authors:** Oxidative Medicine and Cellular Longevity

Oxidative Medicine and Cellular Longevity has retracted the article titled “Biomolecular Modulation of Neurodegenerative Events during Ageing” [1]. As raised on PubPeer, the article was found to contain images with signs of duplication and manipulation in Figures 4 and 5.

- Panels b' and c' in Figure 4 appear to show the same image, but rotated 180 degrees in c' and shown at a shorter exposure
- Panels b' and c' in Figure 5 also appear to show the same image, but rotated 180 degrees in c' and shown at a shorter exposure
- Panel b in Figure 4 and panel b in Figure 5 appear to show an overlap when one of them is rotated 180 degrees [2].

The figure comparisons are shown in the supplementary materials. The authors do not agree with retraction, but we consulted our Editorial Board who confirmed the concern.

## Supplementary Materials

Supplementary Materials Figure duplication in Figures 4 and 5 of OMCL/978654.
