# Supplementary material for: Retracted: Biomolecular Modulation of Neurodegenerative Events during Ageing
Source: Oxid Med Cell Longev. 2019 Oct 15;2019:3571916. doi: 10.1155/2019/3571916 (PMC6816003; doi:10.1155/2019/3571916)
Supplement: Supplementary Materials — Figure duplication in Figures 4 and 5 of OMCL/978654. [file 3571916.f1.docx]

**Figure duplication in Figures 4 and 5 of OMCL/978654**


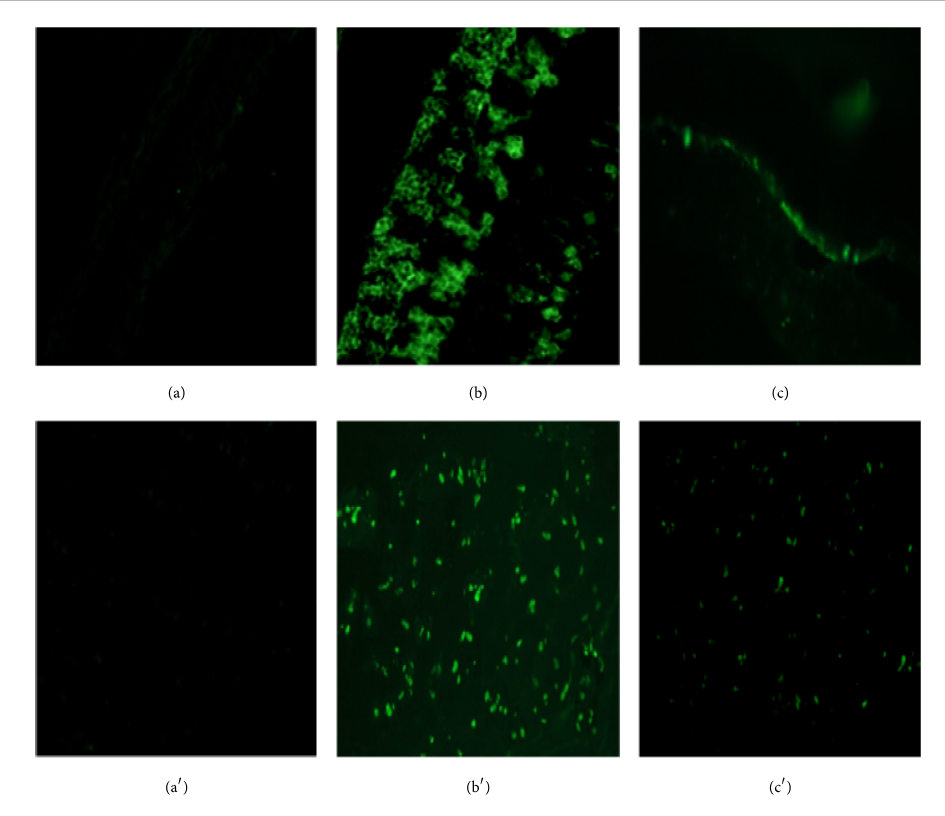


Figure 4: Inducible nitric oxide synthase (iNOS) immunolocalization in the sections of the optic nerve head (a/b/c) and in the retina (a′/b′/c′). ((a) and (a′)) Young rat without iNOS immunolocalization (6 months, negative control). ((b) and (b′)) Aged rat fed normally with high iNOS expression in cells. ((c) and (c′)) Aged rat after oral supplementation of α-lipoic acid (ALA) and superoxide dismutase (SOD) with reduced immunofluorescence, indicating lower iNOS expression.


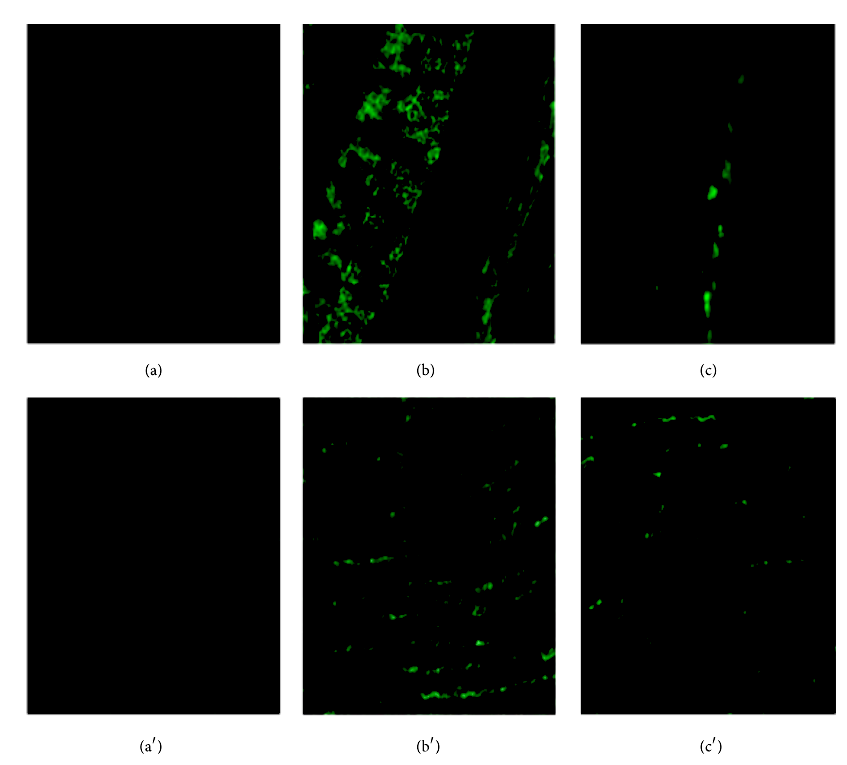


Figure 5: Caspase-3 immunolocalization in the sections of the optic nerve head (a/b/c) and in the retina (a′/b′/c′). ((a) and (a′)) Young rat without caspase-3 immunolocalization (6 months, negative control). ((b) and (b′)) Aged rat fed normally with high caspase-3 expression. ((c) and (c′)) Aged rat after oral supplementation of α-lipoic acid (ALA) and superoxide dismutase (SOD) with reduced immunofluorescence, indicating lower caspase-3 expression.

Figure 5b’, rotated 180 degrees and with increased contrast, appears to be the same as Figure 5c’:


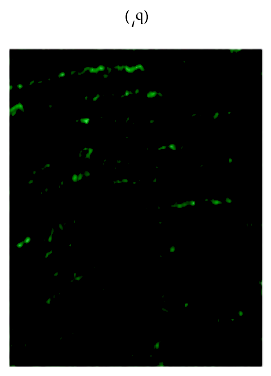

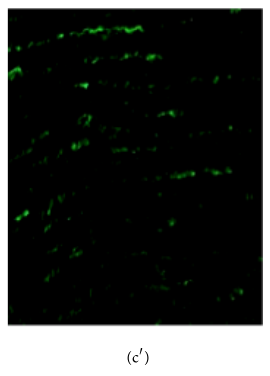


Respectively, they represent (5b’) Caspase-3 immunolocalization in the sections of the retina of aged rat fed normally with high caspase-3 expression, and (5c′) Caspase-3 immunolocalization in the sections of the retina of aged rat after oral supplementation of α-lipoic acid (ALA) and superoxide dismutase (SOD) with reduced immunofluorescence, indicating lower caspase-3 expression.

Figure 5b, rotated 180 degrees, appears to show part of the same image as Figure 4b:


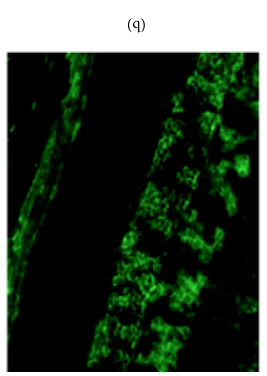

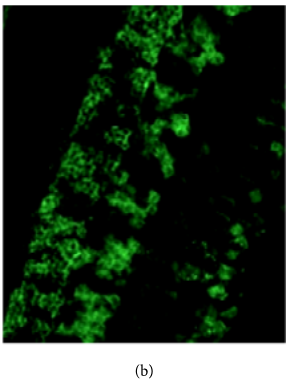


Respectively, they represent (4b) Inducible nitric oxide synthase (iNOS) immunolocalization in the sections of the optic nerve head, aged rat fed normally with high iNOS expression in cells, and (5b) Caspase-3 immunolocalization in the sections of the optic nerve head in aged rat fed normally with high caspase-3 expression.

Figure 4b’, rotated 180 degrees and with increased contrast and reduced exposure, appears to be the same as Figure 4c’:


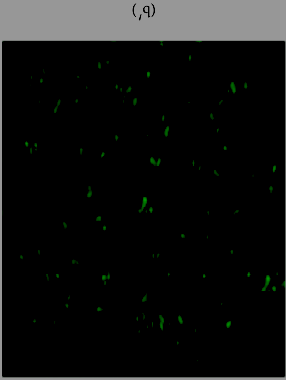

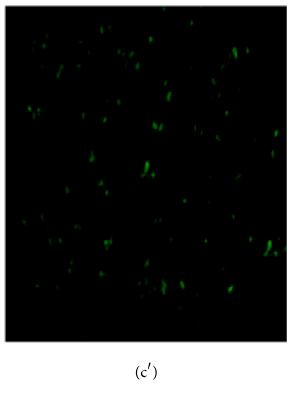


Respectively, they represent (4b’) Inducible nitric oxide synthase (iNOS) immunolocalization in the sections of the retina in aged rat fed normally with high iNOS expression in cells, and (4c’) Inducible nitric oxide synthase (iNOS) immunolocalization in the sections of the retina in aged rat after oral supplementation of α-lipoic acid (ALA) and superoxide dismutase (SOD) with reduced immunofluorescence, indicating lower iNOS expression.
